# Anticoagulant Use as an Independent Risk Factor and Higher In-Hospital Mortality in Patients Showing Alveolar Hemorrhage in Diffuse Lung Disease

**DOI:** 10.3390/medicina57101094

**Published:** 2021-10-13

**Authors:** Motoi Ugajin, Hisanori Kani, Hideo Hattori

**Affiliations:** 1 Department of Respiratory Medicine, Nagoya Tokushukai General Hospital, 2-52 Kouzouji-cho Kita, Kasugai City 487-0016, Japan; 2 Department of Respiratory Medicine and Allergology, Aichi Medical University Hospital, Nagakute City 480-1195, Japan; 3 Department of Thoracic Surgery, Nagoya Tokushukai General Hospital, Kasugai City 487-0016, Japan; k2-hisanori@nagoya.tokushukai.or.jp; 4 Department of Pathology, Nagoya Tokushukai General Hospital, Kasugai City 487-0016, Japan; h-hideo@nagoya.tokushukai.or.jp

**Keywords:** alveolar hemorrhage, diffuse lung disease, bronchoalveolar lavage, anticoagulant, respiratory failure, mortality

## Abstract

*Background and objectives*: Bronchoalveolar lavage (BAL) is commonly performed to evaluate diffuse lung disease and occasionally to identify alveolar hemorrhage. However, the clinical impact of alveolar hemorrhage and its risk factors in patients with diffuse lung disease have not been clarified. *Materials and Methods*: We retrospectively analyzed the medical records of all patients who underwent BAL to evaluate diffuse lung disease from January 2017 to December 2020. Alveolar hemorrhage was defined as progressive hemorrhagic BAL fluid or the presence of ≥20% hemosiderin-laden macrophages in the BAL fluid. Logistic regression analysis was performed to assess the association between alveolar hemorrhage and other factors. *Results*: Sixty subjects were enrolled in this study. Alveolar hemorrhage was observed in 19 subjects (31.7%) with idiopathic interstitial pneumonia, acute respiratory distress syndrome, interstitial pneumonia with autoimmune features, drug-induced lung injury, eosinophilic pneumonia, adenocarcinoma, and systemic lupus erythematosus. The use of anticoagulants was a significant risk factor for alveolar hemorrhage (odds ratio 7.57, *p* = 0.049). Patients with alveolar hemorrhage required intubated mechanical ventilation more frequently (63.2% vs. 24.4%, *p* = 0.005) and had higher in-hospital mortality rates (26.3% vs. 4.9%, *p* = 0.028) than those without alveolar hemorrhage. *Conclusions*: Alveolar hemorrhage was observed in various etiologies. The use of anticoagulants was a significant risk factor for alveolar hemorrhage. Patients with alveolar hemorrhage showed more severe respiratory failure and had higher in-hospital mortality than those without alveolar hemorrhage.

## 1. Introduction

Bronchoalveolar lavage (BAL) is widely performed to evaluate diffuse parenchymal lung disease, and its utility has been documented [1,2]. Progressive hemorrhagic BAL fluid or a high concentration of hemosiderin-laden macrophages in the BAL fluid has been used to confirm alveolar hemorrhage [2,3]. The causes of alveolar hemorrhage vary, and this condition is known to be life-threatening as it is associated with severe respiratory failure [4]. Patients with primary pulmonary hypertension receiving intravenous prostacyclin therapy were prone to show alveolar hemorrhage in the case of combined anticoagulation therapy [5]. Among patients with systemic lupus erythematosus, nephritis, positivity of anti-Sjögren’s syndrome antigen A antibody, thrombocytopenia, and elevation of serum C-reactive protein were reported as significant risk factors of alveolar hemorrhage [6]. However, in patients with diffuse lung disease, the clinical impact of alveolar hemorrhage and its risk factors have not been elucidated. We conducted a single-center, retrospective analysis to evaluate the risk factors and the clinical significance of alveolar hemorrhage in patients with diffuse lung disease.

## 2. Methods

### 2.1. Subjects

The electronic medical records of all patients with diffuse lung disease who underwent BAL at Nagoya Tokushukai General Hospital (a 350-bed teaching hospital, Kasugai city, Aichi prefecture, Japan) between January 2017 and December 2020 were reviewed. Patient characteristics, comorbidities, laboratory findings, chest computed tomography (CT) findings, BAL findings, pathological findings, microbiological findings, and clinical outcomes were extracted from their medical records.

Patients taking anti-hypertensive agents regularly or mentioned as having hypertension on the medical records were considered to have hypertension. Patients with a history of hospitalization due to heart failure or interventional procedure for cardiac disorders (e.g., coronary artery graft surgery, valve replacement surgery, or percutaneous coronary intervention) were considered to have heart disease. Similarly, patients with a history of hospitalization due to brain infarction, brain hemorrhage, or subarachnoid hemorrhage were considered to have cerebrovascular disease, and those with serum creatinine levels ≥1.5 mg/dL or those receiving renal replacement therapy were considered to have kidney disease. Active neoplasms were defined by the presence of antineoplastic agents or the concurrent existence of malignant neoplasms. Two clinical outcomes, namely, patient requirement for intubated mechanical ventilation and in-hospital mortality, were chosen for evaluation.

At our institute, patients with diffuse lung disease routinely undergo the following checkups: (I) chest CT, including high resolution images; (II) blood sampling including cell counts, coagulation tests, biochemistries (C-reactive protein, lactate dehydrogenase, and Krebs von den Lugen-6), and auto-antibodies (rheumatoid factor, anti-nuclear antibody, myeloperoxidase anti-neutrophil cytoplasmic antibody, and proteinase 3 anti-neutrophil cytoplasmic antibody); and (III) urinalysis, including microscopic examination to detect red blood cells in urine. All chest CT images were reviewed by a certified pulmonologist (Motoi Ugajin or Hisanori Kani) as well as an external certified radiologist (Radiolonet Tokai, Nagoya, Japan). Hematuria was defined as the existence of five or more red blood cells per high-powered field in the urine.

The causes of diffuse lung disease were ascertained based on the clinical course of the disease, radiographic findings, serological findings, pathological findings, microbiological findings, and analysis of BAL. For example, eosinophilic pneumonia was diagnosed when BAL included >25% eosinophil differential count or lung specimens showed accumulation of eosinophils [2,7]. Interstitial pneumonia with autoimmune features was defined according to the official European Respiratory Society/American Thoracic Society research statement [8]. Acute respiratory distress syndrome was defined based on the Berlin definition [9].

The requirement of informed consent was waived because of the retrospective nature of the study, and the study protocol was approved by the research ethics committee of the Tokushukai group (approval number: TGE01375-016).

### 2.2. Procedure of BAL and Definition of Alveolar Hemorrhage

**Figure 1:**
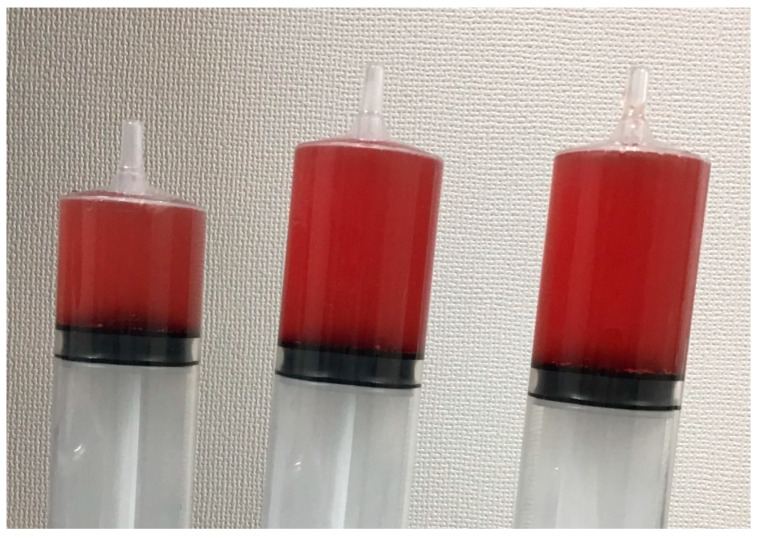
An example of hemorrhagic bronchoalveolar lavage fluid.

BAL was performed as follows: The bronchoscope was placed in a wedged position. Fifty milliliters of normal saline was instilled and then gently retrieved using a syringe with hand suction through the bronchoscope. This process was repeated three times. The retrieved fluid was evaluated for differential cell counts, bacterial cultures including acid-fast bacilli, and cytological examination including iron staining. Alveolar hemorrhage was defined as progressive hemorrhagic BAL fluid (Figure 1) or the presence of ≥20% hemosiderin-laden macrophages in the BAL fluid [2,3]. When the amount of retrieved fluid was less than 20% of the instilled amount, the BAL was considered inadequate and was excluded from the study. If BAL was repeated twice or more in the same patient, only the first BAL was included in the study.

### 2.3. Statistical Analysis

Data are expressed as the number (%) or median (25th to 75th percentile range). Differences between the groups were tested using the Mann–Whitney *U* test for continuous variables and Fisher’s exact test for categorical variables. Logistic regression analysis was performed to assess the association between alveolar hemorrhage and patient characteristics as well as laboratory findings. Results with *p* < 0.2 in the univariate analysis were included in the logistic regression analysis. Results with a two-tailed probability value less than 0.05 were considered statistically significant. All statistical analyses were performed using Ekuseru-Tokei 2012 (Social Survey Research Information Co., Ltd., Tokyo, Japan).

## 3. Results

### 3.1. Study Cohort

Seventy-three BALs were performed in 66 patients to evaluate diffuse lung disease from January 2017 to December 2020. Of these, 60 subjects were included in the study. Six subjects showed insufficient amount of BAL fluid and BAL was repeated in seven subjects.

The causes of diffuse lung disease are listed in Table 1. Alveolar hemorrhage was observed in 19 (31.7%) patients (idiopathic interstitial pneumonia, *n* = 8; acute respiratory distress syndrome, *n* = 3; interstitial pneumonia with autoimmune features, *n* = 3; drug-induced lung injury, *n* = 1; eosinophilic pneumonia, *n* = 1; adenocarcinoma, *n* = 1; systemic lupus erythematosus, *n* = 1; and unknown etiology, *n* = 1). Fifteen out of 19 (78.9%) patients with alveolar hemorrhage showed the presence of ≥20% hemosiderin-laden macrophages in the BAL fluid, while the remaining 4 (21.0%) patients showed less than 20% hemosiderin-laden macrophages in the BAL fluid in spite of apparent hemorrhagic BAL fluid. Alveolar hemorrhage was not observed in 41 (68.3%) patients.

### 3.2. Patient Characteristics and Laboratory Findings

Table 2 shows the comparison of demographic characteristics and laboratory findings between patients with and without alveolar hemorrhage. Patients with alveolar hemorrhage were older (median: 80 vs. 74 years, *p* = 0.023) and used antiplatelet agents (42.1% vs. 14.6%, *p* = 0.046) and anticoagulants (52.6% vs. 9.8%, *p* < 0.001) more frequently than those without alveolar hemorrhage. Patients with alveolar hemorrhage showed higher serum C-reactive protein levels (10.76 vs. 4.40 mg/dL, *p* = 0.026), lower hemoglobin levels (10.1 vs. 12.7 g/dL, *p* = 0.008), and higher prothrombin time-international normal ratio (1.27 vs. 1.14, *p* = 0.008) than those without alveolar hemorrhage.

Logistic regression analysis of patient characteristics and laboratory findings revealed that anticoagulant use was a significant risk factor for alveolar hemorrhage (odds ratio 7.57; *p* = 0.049; Table 3).

### 3.3. BAL Findings and Clinical Outcomes

Table 4 outlines the comparison of the BAL findings and clinical outcomes between patients with and without alveolar hemorrhage. Patients with alveolar hemorrhage showed a lower proportion of lymphocytes (46% vs. 62%, *p* = 0.015) and a higher proportion of neutrophils (31% vs. 16%, *p* = 0.005) than those without alveolar hemorrhage.

Twenty-two (36.7%) patients required intubated mechanical ventilation. Among these 22 intubated patients, nine patients underwent BAL on the day of intubation, 10 patients underwent BAL on the day after intubation, two patients underwent BAL two days after intubation, and one patient was intubated nine days after BAL. Seven patients (11.7%) died during their hospital stay due to respiratory failure, and all of these deceased patients underwent intubated mechanical ventilation. Patients with alveolar hemorrhage required intubated mechanical ventilation more frequently (63.2% vs. 24.4%, *p* = 0.005) and had higher in-hospital mortality rates (26.3% vs. 4.9%, *p* = 0.028) than those without alveolar hemorrhage. 

## 4. Discussion

The present study showed that alveolar hemorrhage of various etiologies was observed in patients with diffuse lung disease. Further, the use of anticoagulants was a significant risk factor for alveolar hemorrhage. Finally, patients with alveolar hemorrhage showed more severe respiratory failure and higher in-hospital mortality than those without alveolar hemorrhage.

One of the major causes of alveolar hemorrhage is injury to the alveolar microcirculation [4]. Idiopathic interstitial pneumonias, especially idiopathic pulmonary fibrosis (IPF), cause structural changes in alveolar microcirculation through endothelial cell apoptosis and capillary loss [10]. In fact, patients with IPF showed a significantly higher density of hemosiderin-laden macrophages in their BAL fluids than healthy smokers [11]. Previous studies on lung disease have reported cases of alveolar hemorrhage in patients with drug-induced lung injury, pneumocystis pneumonia, and influenza viral infection [12,13,14]. Maldonado et al. reported that one-third of the patients surgically proven to have diffuse alveolar damage showed BAL fluids consisting of ≥20% hemosiderin-laden macrophages [15]. In summary, previous studies have demonstrated that alveolar hemorrhage can be observed in various lung diseases; these results are in accordance with our findings.

In our study cohort, more than one-third of patients required intubated mechanical ventilation due to severe respiratory failure. Almost all of these patients underwent BAL after intubation. Therefore, we could not exclude the possibility of injury to the alveolar microcirculation caused by mechanical ventilation. However, in our institute, both low tidal volume and plateau pressure less than 30 cm H_2_O were generally applied to mechanical ventilation setting in order to avoid ventilator-induced lung injury [16]. Moreover, although we performed BAL at least within two days after the initiation of intubated mechanical ventilation, most of these intubated patients with alveolar hemorrhage showed the presence of ≥20% hemosiderin-laden macrophages in their BAL fluids. The accumulation of hemosiderin-laden macrophage requires at least 48 h after intra-alveolar bleeding [4]. This means that most patients in the present study had alveolar hemorrhage before the initiation of intubated mechanical ventilation.

We found that anticoagulant use was an independent risk factor for alveolar hemorrhage in patients with diffuse lung disease. To the best of our knowledge, there are 14 case reports about the occurrence of alveolar hemorrhage during warfarin therapy. In contrast, only one case report mentioned the occurrence of alveolar hemorrhage during direct oral anticoagulant therapy [17]. Direct oral anticoagulants are generally considered safer than warfarin with respect to the risk of major bleeding events [18,19]. However, among elderly patients aged ≥ 75 years, the use of direct oral anticoagulants was associated with more frequent major bleeding events than warfarin use [20]. Moreover, a study using mice infected with influenza A virus showed that both warfarin and dabigatran etexilate, a direct oral anticoagulant, equally increased the risk of alveolar hemorrhage [21]. In the present study, of the 10 patients with alveolar hemorrhage during anticoagulation therapy, three patients used warfarin, while the remaining seven patients used direct oral anticoagulants (edoxaban, *n* = 6; and apixaban, *n* = 1). Among the four patients without alveolar hemorrhage during anticoagulation therapy, three patients used warfarin and one patient used apixaban. It remains unclear which anticoagulation therapy is safer in terms of alveolar hemorrhage, and further investigations are required.

Combined antiplatelet-anticoagulant therapy was applied to such patients as having cardiovascular disease with atrial fibrillation [22]. In the present study, five patients took combined antiplatelet-anticoagulant therapy. Three patients showed alveolar hemorrhage (aspirin + edoxaban, *n* = 2; and clopidogrel + edoxaban, *n* = 1), while the remaining two patients did not show alveolar hemorrhage (aspirin + warfarin, *n* = 2). Higher incidence of bleeding events is a concern for combined antiplatelet-anticoagulant therapy compared with antiplatelet or anticoagulant monotherapy. In fact, aspirin plus warfarin therapy was reported to increase the risk of bleeding events as compared to warfarin monotherapy in patients with atrial fibrillation [23]. However, several recent studies showed that combined antiplatelet-anticoagulant therapy did not increase the risk of bleeding as compared to anticoagulant monotherapy [24,25,26]. In the present study, we could not assess the influence of combined antiplatelet-anticoagulant therapy for alveolar hemorrhage because there were a limited number of patients taking combined antiplatelet-anticoagulant therapy in our study cohort. Concerning the relationship between combined antiplatelet-anticoagulant therapy and alveolar hemorrhage, further investigations are needed.

We found that patients with alveolar hemorrhage showed more severe respiratory failure and higher in-hospital mortality than those without alveolar hemorrhage. In the present study, 12 out of 19 patients (63.2%) with alveolar hemorrhage required intubated mechanical ventilation, and five patients (26.3%) died during their hospital stay. This mortality rate was similar to that reported in a previous study by Prost et al., which included 97 patients with diffuse alveolar hemorrhage [27]. Another study performed in an intensive care unit reported that 86% of the patients with diffuse alveolar hemorrhage required intubated mechanical ventilation, and the in-hospital mortality rate was around 51% [28]. Furthermore, in patients surgically proven to have diffuse alveolar damage, significantly higher mortality was observed in the case of the concurrent existence of alveolar hemorrhage [15]. In accordance with the result of the present study, the main cause of mortality in patients with alveolar hemorrhage is reported to be acute respiratory failure rather than complications associated with immunosuppressive therapy [29]. In short, alveolar hemorrhage itself is a life-threatening condition causing severe respiratory failure.

We must note that the present study had at least two limitations. First, it may lack sufficient histological examination to evaluate diffuse lung disease because surgical lung biopsy is not routinely performed. Many patients included in our study were not suitable candidates for surgical lung biopsy because of respiratory failure, cognitive impairment, and other comorbidities. Instead, diffuse lung disease was diagnosed through a detailed review of medical records and was based on discussions among three certified clinicians, two pulmonologists and a pathologist. Second, our study included a small number of patients as it was a single-center study. To reinforce the clinical significance of alveolar hemorrhage in diffuse lung disease, a multicenter study including a large number of subjects is required.

In conclusion, our study in patients with diffuse lung disease revealed alveolar hemorrhage of various etiologies. We also found that the use of anticoagulants was a significant risk factor for alveolar hemorrhage. Furthermore, patients with alveolar hemorrhage showed more severe respiratory failure and higher in-hospital mortality than those without alveolar hemorrhage.

## Figures and Tables

**Table 1:** Causes of diffuse lung disease.

Cause	Number of Patients (Alveolar Hemorrhage)
Idiopathic interstitial pneumonia	25 (8)
Acute respiratory distress syndrome	7 (3)
Interstitial pneumonia with autoimmune features	6 (3)
Drug-induced lung injury	5 (1)
Eosinophilic pneumonia	5 (1)
Hypersensitivity pneumonitis	4 (0)
Adenocarcinoma	2 (1)
Sarcoidosis	2 (0)
Systemic lupus erythematosus	1 (1)
Pneumocystis pneumonia	1 (0)
Systemic sclerosis	1 (0)
Unknown	1 (1)
Total	60 (19)

**Table 2:** Demographic characteristics and laboratory findings in patients with and without alveolar hemorrhage.

	Hemorrhage (*n* = 19)	Non-Hemorrhage (*n* = 41)	*p* Value
Characteristics			
Age (years)	80 (75–82)	74 (61–80)	0.023
Males	15 (78.9)	27 (65.8)	0.375
Use of antiplatelet agents	8 (42.1)	6 (14.6)	0.046
Use of anticoagulants	10 (52.6)	4 (9.8)	<0.001
Comorbidities			
Hypertension	13 (68.4)	21 (51.2)	0.268
Heart disease	8 (42.1)	8 (19.5)	0.114
Cerebrovascular disease	3 (15.8)	2 (4.9)	0.314
Kidney disease	5 (26.3)	5 (12.2)	0.263
Active neoplasms	0	4 (9.8)	0.297
Laboratory findings			
C-reactive protein (mg/dL)	10.76 (5.27–13.00)	4.40 (0.71–9.22)	0.026
Lactate dehydrogenase (U/L)	278 (223–347)	243 (202–341)	0.325
Krebs von den Lungen-6 (U/mL)	349 (291–552)	586 (263–1397)	0.282
Hemoglobin (g/dL)	10.1 (9.0–12.9)	12.7 (11.2–13.6)	0.008
Platelet count (×10^4^/uL)	20.2 (14.4–24.9)	24.0 (18.2–30.0	0.107
PT-INR	1.27 (1.17–1.47)	1.14 (1.05–1.22)	0.008
APTT (sec)	31.7 (28.8–35.1)	30.5 (28.2–32.6)	0.23
Hematuria	4 (21.1)	4 (9.8)	0.249

Data are expressed as the number (%) or median (25th–75th range). PT-INR: prothrombin time- international normal ratio, APTT: activated partial thromboplastin time.

**Table 3:** Logistic regression analysis for alveolar hemorrhage.

	Odds Ratio	95% Confidence Interval	*p* Value
Age	1.05	0.97–1.14	0.197
Use of antiplatelet agents	2.02	0.33–12.4	0.447
Use of anticoagulants	7.57	1.01–56.8	0.049
Heart disease	0.81	0.14–4.58	0.808
C-reactive protein (mg/dL)	1.06	0.97–1.17	0.192
Hemoglobin (g/dL)	0.86	0.57–1.30	0.476
Platelet count (×10^4^ /uL)	1.00	0.93–1.09	0.922
PT-INR	0.79	0.12–5.25	0.810

PT-INR: prothrombin time-international normal ratio.

**Table 4:** Bronchoalveolar lavage and outcomes in subjects with or without alveolar hemorrhage.

	Hemorrhage (*n* = 19)	Non-Hemorrhage (*n* = 41)	*p* Value
Bronchoalveolar lavage			
Recovery rate (%)	40 (35–47)	33 (27–47)	0.136
Total cell count (×10^5^/mL)	9.8 (6.0–27.7)	8.8 (3.4–24.0)	0.365
Macrophage (%)	8 (4–11)	7 (4–14)	0.824
Lymphocyte (%)	46 (26–56)	62 (40–75)	0.015
Neutrophil (%)	31 (17–60)	16 (11–25)	0.005
Eosinophil (%)	2 (1–4)	2 (1–5)	0.891
Clinical outcome			
Intubated ventilation	12 (63.2)	10 (24.4)	0.005
In-hospital mortality	5 (26.3)	2 (4.9)	0.028

Data are expressed as the number (%) or median (25th–75th range).

## Data Availability

The presented date in this study are available on request from the corresponding author. The data are not publicly available due to ethical restrictions.

## References

[1] Wells A.U. (2010). The clinical utility of bronchoalveolar lavage in diffuse parenchymal lung disease. Eur. Respir. Rev..

[2] Meyer K.C., Raghu G., Baughman R.P., Brown K.K., Costabel U., du Bois R.M., Drent M., Haslam P.L., Kim D.S., Nagai S. (2012). An official American Thoracic Society clinical practice guideline: The clinical utility of bronchoalveolar lavage cellular analysis in interstitial lung disease. Am. J. Respir. Crit. Care Med..

[3] De Lassence A., Fleury-Feith J., Escudier E., Beaune J., Bernaudin J.F., Cordonnier C. (1995). Alveolar hemorrhage. Diagnostic criteria and results in 194 immunocompromised hosts. Am. J. Respir. Crit. Care Med..

[4] Lara A.R., Schwarz M.I. (2010). Diffuse alveolar hemorrhage. Chest.

[5] Ogawa A., Matsubara H., Fujio H., Miyaji K., Nakamura K., Morita H., Saito H., Kusano K.F., Emori T., Date H. (2005). Risk of alveolar hemorrhage in patients with primary pulmonary hypertension--anticoagulation and epoprostenol therapy. Circ. J..

[6] Sun Y., Zhou C., Zhao J., Wang Q., Xu D., Zhang S., Shen M., Hou Y., Tian X., Li M. (2020). Systemic lupus erythematosus-associated diffuse alveolar hemorrhage: A single-center, matched case-control study in China. Lupus.

[7] Jeong Y.J., Kim K.I., Seo I.J., Lee C.H., Lee K.N., Kim K.N., Kim J.S., Kwon W.J. (2007). Eosinophilic lung diseases: A clinical, radiologic, and pathologic overview. Radiographics.

[8] Fischer A., Antoniou K.M., Brown K.K., Cadranel J., Corte T.J., Du Bois R.M., Lee J.S., Leslie K.O., Lynch D.A., Matteson E.L. (2015). An official European Respiratory Society/American Thoracic Society research statement: Interstitial pneumonia with autoimmune features. Eur. Respirat. J..

[9] Ranieri V.M., Rubenfeld G.D., Thompson B.T., Ferguson N.D., Caldwell E., Fan E., Camporota L., Slutsky A.S. (2012). ARDS Definition Task Force: Acute respiratory distress syndrome: The Berlin Definition. JAMA.

[10] Farkas L., Kolb M. (2011). Pulmonary microcirculation in interstitial lung disease. Proc. Am. Thorac. Soc..

[11] Puxeddu E., Comandini A., Cavalli F., Pezzuto G., D’Ambrosio C., Senis L., Paci M., Curradi G., Sergiacomi G.L., Saltini C. (2014). Iron laden macrophages in idiopathic pulmonary fibrosis: The telltale of occult alveolar hemorrhage?. Pulm. Pharmacol. Ther..

[12] Iskandar S.B., Abi-Saleh B., Keith R.L., Byrd R.P., Roy T.M. (2006). Amiodarone-induced alveolar hemorrhage. South Med. J..

[13] Tamaki Y., Higa F., Tasato D., Nakamura H., Uechi K., Tamayose M., Haranaga S., Yara S., Tateyama M., Fujita J. (2011). Pneumocystis jirovecii pneumonia and alveolar hemorrhage in a pregnant woman with human T cell lymphotropic virus type-1 infection. Intern. Med..

[14] Gilbert C.R., Vipul K., Baram M. (2010). Novel H1N1 influenza A viral infection complicated by alveolar hemorrhage. Respir. Care..

[15] Maldonado F., Parambil J.G., Yi E.S., Decker P.A., Ryu J.H. (2009). Haemosiderin-laden macrophages in the bronchoalveolar lavage fluid of patients with diffuse alveolar damage. Eur. Respir. J..

[16] Papazian L., Aubron C., Brochard L., Chiche J.D., Combes A., Dreyfuss D., Forel J.M., Guérin C., Jaber S., Mekontso-Dessap A. (2019). Formal guidelines: Management of acute respiratory distress syndrome. Ann. Intensive Care.

[17] Nitta K., Imamura H., Yashio A., Kashima S., Mochizuki K. (2016). Diffuse Alveolar Hemorrhage Associated with Edoxaban Therapy. Case Rep. Crit. Care.

[18] Granger C.B., Alexander J.H., McMurray J.J., Lopes R.D., Hylek E.M., Hanna M., Al-Khalidi H.R., Ansell J., Atar D., Avezum A. (2011). ARISTOTLE Committees and Investigators. Apixaban versus warfarin in patients with atrial fibrillation. N. Engl. J. Med..

[19] Vinogradova Y., Coupland C., Hill T., Hippisley-Cox J. (2018). Risks and benefits of direct oral anticoagulants versus warfarin in a real world setting: Cohort study in primary care. BMJ.

[20] Wong J.M., Maddox T.M., Kennedy K., Shaw R.E. (2020). Comparing Major Bleeding Risk in Outpatients With Atrial Fibrillation or Flutter by Oral Anticoagulant Type (from the National Cardiovascular Disease Registry’s Practice Innovation and Clinical Excellence Registry). Am. J. Cardiol..

[21] Tatsumi K., Antoniak S., Subramaniam S., Gondouin B., Neidich S.D., Beck M.A., Mickelson J., Monroe D.M., Bastarache J.A., Mackman N. (2016). Anticoagulation increases alveolar hemorrhage in mice infected with influenza A. Physiol. Rep..

[22] Ruiz-Nodar J.M., Marín F., Hurtado J.A., Valencia J., Pinar E., Pineda J., Gimeno J.R., Sogorb F., Valdés M., Lip G.Y. (2008). Anticoagulant and antiplatelet therapy use in 426 patients with atrial fibrillation undergoing percutaneous coronary intervention and stent implantation implications for bleeding risk and prognosis. J. Am. Coll. Cardiol..

[23] Flaker G.C., Gruber M., Connolly S.J., Goldman S., Chaparro S., Vahanian A., Halinen M.O., Horrow J., Halperin J.L., SPORTIF Investigators (2006). Risks and benefits of combining aspirin with anticoagulant therapy in patients with atrial fibrillation: An exploratory analysis of stroke prevention using an oral thrombin inhibitor in atrial fibrillation (SPORTIF) trials. Am. Heart J..

[24] Donzé J., Rodondi N., Waeber G., Cornuz J., Aujesky D. (2013). Major bleeding risk in anticoagulated patients receiving concomitant antiplatelet therapy: A prospective study. Thromb. Res..

[25] Chen P.C., Lip G.Y., Yeh G., Lin H.J., Chien K.L. (2015). Risk of bleeding and stroke with oral anticoagulation and antiplatelet therapy in patients with atrial fibrillation in Taiwan: A nationwide cohort study. PLoS ONE.

[26] Tinkham T.T., Vazquez S.R., Jones A.E., Witt D.M. (2020). Direct oral anticoagulant plus antiplatelet therapy: Prescribing practices and bleeding outcomes. J. Thromb. Thrombol..

[27] de Prost N., Parrot A., Picard C., Ancel P.Y., Mayaud C., Fartoukh M., Cadranel J. (2010). Diffuse alveolar haemorrhage: Factors associated with in-hospital and long-term mortality. Eur. Respir. J..

[28] Rabe C., Appenrodt B., Hoff C., Ewig S., Klehr H.U., Sauerbruch T., Nickenig G., Tasci S. (2010). Severe respiratory failure due to diffuse alveolar hemorrhage: Clinical characteristics and outcome of intensive care. J. Crit. Care..

[29] Afessa B., Tefferi A., Litzow M.R., Peters S.G. (2002). Outcome of diffuse alveolar hemorrhage in hematopoietic stem cell transplant recipients. Am. J. Respir Crit. Care Med..

